# Bloody Nipple Discharge in a Man with Benign Papillomas: A Case Report

**DOI:** 10.7759/cureus.4431

**Published:** 2019-04-11

**Authors:** Emma G MacInnes, Isobel Haigh, Anju Nijhawan, Brian Hogan

**Affiliations:** 1 Breast Surgery, Leeds Teaching Hospitals Trust, Leeds, GBR; 2 Breast Radiology, Leeds Teaching Hospitals Trust, Leeds, GBR; 3 Pathology, Leeds Teaching Hospitals Trust, Leeds, GBR

**Keywords:** nipple, discharge, papilloma, benign

## Abstract

Blood-stained nipple discharge in a man would usually be proved to be male breast cancer. We present a case where this unusual presentation was associated with benign intraductal papillomas, managed with simple duct excision, preserving the patient’s nipple whilst adequately investigating and managing the condition.

## Introduction

Blood-stained or other abnormal nipple discharge is associated with malignancy in 3% of women [1], with a higher rate anticipated in men presenting with this symptom. If it is associated with a palpable mass or abnormal breast imaging the rate of underlying malignancy increases. Whilst carcinoma and in situ carcinoma are possible there are other benign explanations for abnormal discharge, including physiological discharge, duct ectasia and intraductal papillomas. Cytological assessment of discharge has been demonstrated to lack sensitivity and specificity to allow it to be used to make a diagnosis and normal imaging does not exclude malignancy [2]. Duct excision is the standard of care for women with persisting spontaneous, unilateral nipple discharge to exclude an occult malignant process [3].

Intraductal papillomas are high-risk precursor lesions, with potential to progress to atypia, in situ and invasive carcinomas, with an increased risk also present in the immediately surrounding tissue and a breast cancer risk twice that of the general population [4], even after excision [5]. If needle core biopsy demonstrates a seemingly benign intraductal papilloma, it should be noted that up to 10.7% are upgraded with atypia and malignancy subsequently identified on surgical excision specimens [6]. With the presence of atypia on core biopsy, 32% are subsequently upgraded to malignancy by surgical excision [7]. Total duct excision, for centrally sited, retroareolar papillomas, is generally well tolerated though carries the risk of nipple necrosis, nipple inversion and the loss of nipple sensation.

## Case presentation

We present a 62-year-old male patient, who presented with a few months of left-sided, episodic, blood-stained nipple discharge, occurring spontaneously and without warning. The discharge had the appearance of ‘frank blood’. He had not noticed any other changes to the breast or nipple and had no history of trauma. His previous history included a mild inflammatory arthritis for which he no longer required medication and a microprolactinoma, diagnosed 13 years previously, for which he required testosterone supplementation due to hypogonadotropic hypogonadism, but no other treatment. At the time of the presentation with blood stained nipple discharge, prolactin levels were within normal ranges as were other hormone assays related to his pituitary function. Cabergoline had been discontinued four years prior to presentation. There was no family history of breast cancer, he did not smoke or take alcohol in excess.

**Figure 1:**
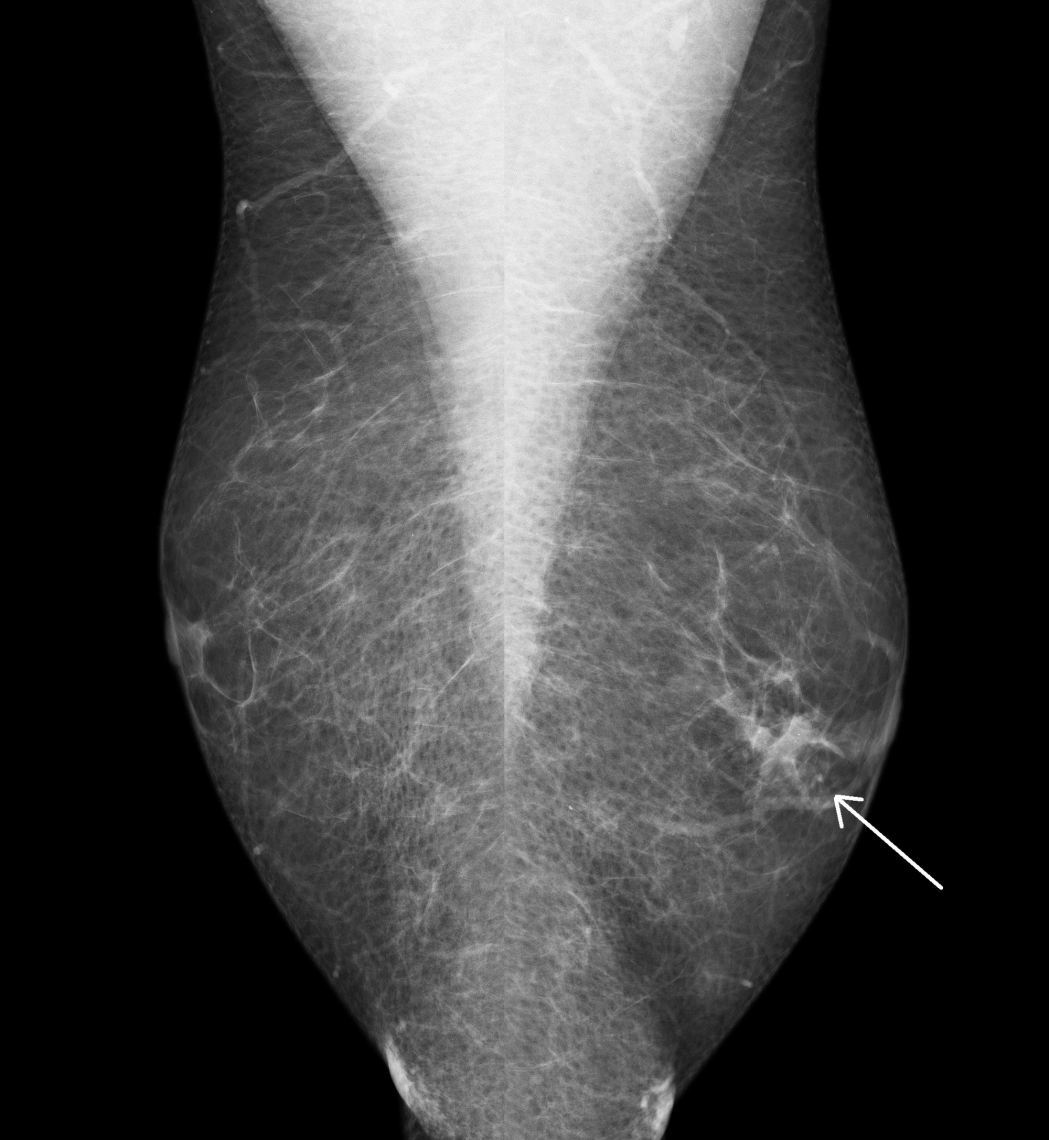
Mammography

Clinical assessment found no visible or palpable abnormality in the breast disc but demonstrated the bloody discharge. Ultrasonography demonstrated subareolar ducts but no focal mass. Mammography was normal on the right but indeterminate on the left with a 16-mm asymmetry in the 9-o-clock position within the retroareolar tissue and with associated flecks of benign appearing calcification (Figure 1). Cytology of a smear of discharge showed plentiful red blood cells and histiocytes with no epithelial cells present. As duct excision was planned, core biopsy was not performed.

**Figure 2:**
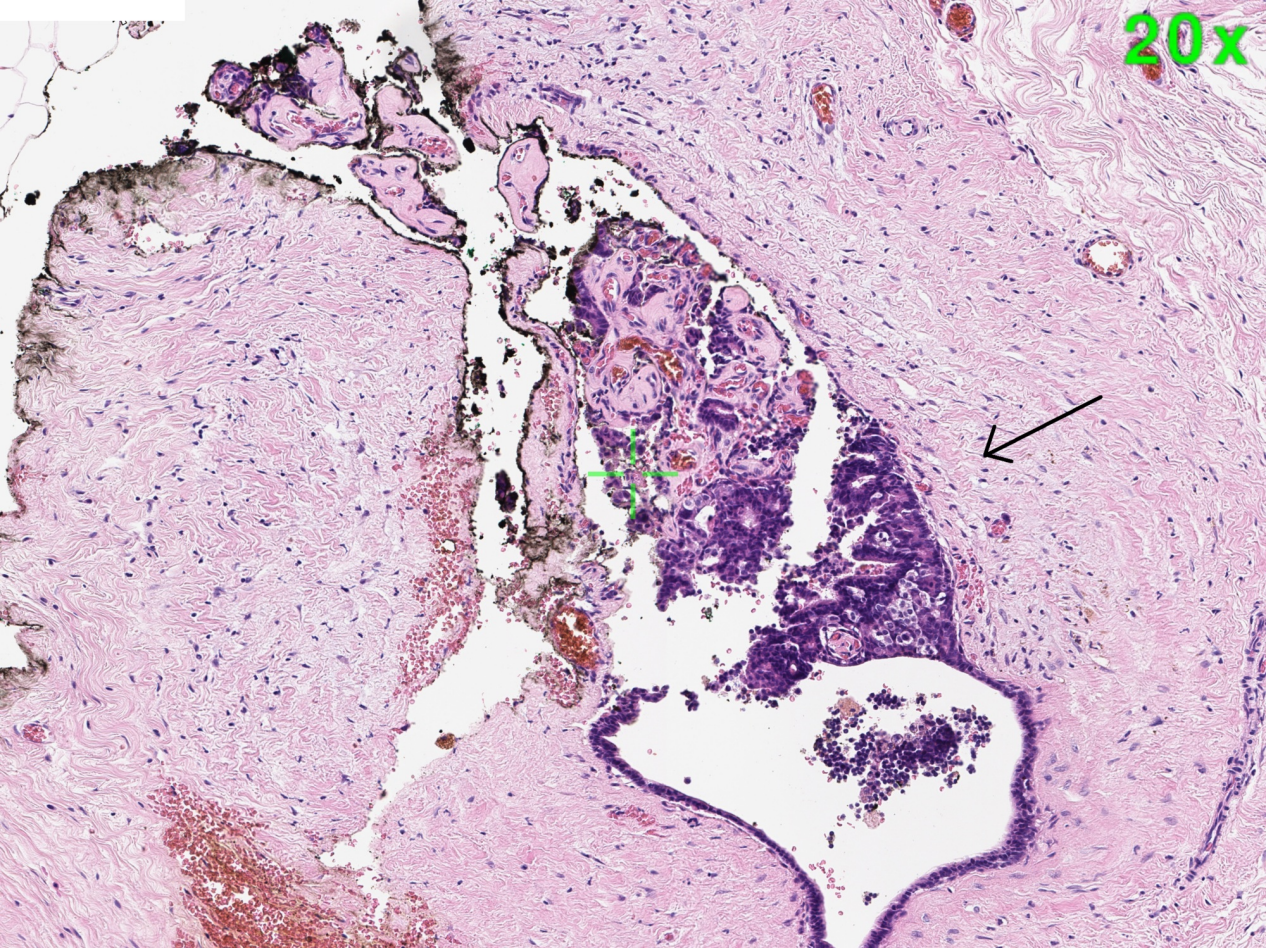
Histopathology image of partially sclerosed intraductal papilloma

The case was discussed in a multi-disciplinary team meeting and a left total duct excision was recommended. This was performed under general anaesthetic, with an anterior shave of tissue taken from behind the nipple. Pathological findings were unusual in the context of a male patient, showing a few scattered benign intraductal papillomas measuring up to 2 mm (Figure 2). Immunohistochemistry showed heterogenous positivity with cytokeratin (CK) 5/6. No atypia was noted. The patient made an uneventful recovery with no further reported nipple discharge and preservation of his nipple and chest wall symmetry. No surveillance is planned.

## Discussion

Benign intraductal papillomas in men are rare but may be the cause of blood-stained nipple discharge. Management is not gender dependent and involves clinical, radiological and pathological assessment, often requiring open surgical excision of the retroareolar tissue. The role of cytological assessment of the discharge in men is unclear and it should not be used to avoid more invasive forms of biopsy. If cytology demonstrates malignancy, however, treatment could proceed directly to mastectomy potentially avoiding the need for two operations. The tendency to assume that nipple symptoms in men are due to malignancy could equally have resulted in disfiguring overtreatment. The risk of future malignancy in men with breast papillomatosis is not known. Whether there is a role for surveillance, particularly in cases with atypia, is unclear but this could be considered.

## Conclusions

Men presenting with bloody nipple discharge must be assumed to have breast malignancy until proven otherwise. This case does, however, illustrate the fact that anything from the spectrum of benign breast disorders seen in women can also be seen, albeit rarely, in men. Performing a mastectomy would have been significant overtreatment of a benign pathology. Duct excision can be safely performed in men and provides an opportunity to preserve the aesthetics of the male breast whilst investigating the cause of and treating suspicious nipple discharge.
